# Transcranial direct-current stimulation induced in stroke patients with aphasia: a prospective experimental cohort study

**DOI:** 10.1590/1516-3180.2013.1316595

**Published:** 2013-12-01

**Authors:** Michele Devido Santos, Rubens José Gagliardi, Ana Paula Machado Goyano Mac-Kay, Paulo Sergio Boggio, Roberta Lianza, Felipe Fregni

**Affiliations:** I BSc, MD, PhD. Professor of Speech Pathology and Audiology, Faculdade de Ciências Médicas, Santa Casa de São Paulo, São Paulo, Brazil.; II MD, PhD. Full Professor and Head of Discipline of Neurology, Faculdade de Ciências Médicas, Santa Casa de São Paulo, São Paulo, Brazil.; III BSc, PhD. Professor of Speech Pathology and Audiology, Faculdade de Ciências Médicas, Santa Casa de São Paulo, São Paulo, Brazil.; IV BSc, PhD. Professor of Cognitive Neuroscience, Cognitive Neuroscience Laboratory, Mackenzie Presbyterian University, São Paulo, Brazil.; V PT. Physiotherapist, Irmandade Santa Casa de Misericórdia de São Paulo, São Paulo, Brazil.; VI MD, PhD. Professor of Neurology, Laboratory of Neuromodulation, Spaulding Rehabilitation Hospital, Harvard Medical School; and Head of Neuromodulation Laboratory, Berenson-Allen Center for Noninvasive Brain Stimulation, Beth Israel Deaconess Medical Center, Harvard Medical School, Boston, Massachusetts, United States.

**Keywords:** Aphasia, Stroke, Electric stimulation, Speech disorders, Language disorders, Afasia, Acidente vascular cerebral, Estimulação elétrica, Distúrbios da fala, Transtornos da linguagem

## Abstract

**CONTEXT AND OBJECTIVE:**

Previous animal and human studies have shown that transcranial direct current stimulation can induce significant and lasting neuroplasticity and may improve language recovery in patients with aphasia. The objective of the study was to describe a cohort of patients with aphasia after stroke who were treated with transcranial direct current stimulation.

**DESIGN AND SETTING:**

Prospective cohort study developed in a public university hospital.

**METHODS:**

Nineteen patients with chronic aphasia received 10 transcranial direct current stimulation sessions lasting 20 minutes each on consecutive days, using a current of 2 mA. The anode was positioned over the supraorbital area and the cathode over the contralateral motor cortex. The following variables were analyzed before and after the 10 neuromodulation sessions: oral language comprehension, copying, dictation, reading, writing, naming and verbal fluency.

**RESULTS:**

There were no adverse effects in the study. We found statistically significant differences from before to after stimulation in relation to simple sentence comprehension (P = 0.034), naming (P = 0.041) and verbal fluency for names of animals (P = 0.038). Improved scores for performing these three tasks were seen after stimulation.

**CONCLUSIONS:**

We observed that excitability of the primary motor cortex through transcranial direct current stimulation was associated with effects on different aspects of language. This can contribute towards future testing in randomized controlled trials.

## Abstract (pt)

**CONTEXTO E OBJETIVO:**

Estudos prévios em animais e humanos mostram que a estimulação transcraniana por corrente contínua pode induzir neuroplasticidade significante e duradoura e pode melhorar a recuperação de linguagem na afasia. O objetivo do estudo foi descrever uma coorte de pacientes com afasia após acidente vascular cerebral que foi tratada com estimulação transcraniana por corrente contínua.

**TIPO DO ESTUDO E LOCAL:**

Estudo de coorte único prospectivo realizado em um hospital público universitário.

**MÉTODO:**

Dezenove pacientes com afasia crônica receberam 10 sessões de estimulação transcraniana por corrente contínua com duração de 20 minutos de cada, corrente de 2 mA em dias consecutivos (anodo posicionado em área supraorbital e catodo no cortes motor contraleral). Foram analisadas as seguintes variáveis antes e depois de 10 sessões de neuromodulação: compreensão de linguagem oral, cópia, ditado, leitura, escrita, nomeação e fluência verbal.

**RESULTADOS:**

Não houve efeitos adversos no estudo. Encontramos diferença estatisticamente significante pré e pós-estimulação para compreensão de frases simples (P = 0.034), nomeação (P = 0.041) e fluência verbal para nomes de animais (P = 0.038). Houve melhora no desempenho em três tarefas após estimulação.

**CONCLUSÃO:**

Observamos que a excitabilidade no córtex motor primário através de estimulação transcraniana por corrente contínua está associada a efeitos em diferentes aspectos da linguagem, além de contribuir para futuras testagens em estudos randomizados.

## INTRODUCTION

Recent evidence has suggested that techniques for noninvasive brain stimulation such as transcranial magnetic stimulation and transcranial direct current stimulation might be beneficial tools for improving language skills among patients with aphasia.^1^ ^,^ ^2^ Although the initial studies were mainly conducted using transcranial magnetic stimulation, new data have shown that transcranial direct current stimulation is a technique that induces significant effects on neuronal spontaneous activity. Because transcranial direct current stimulation has the ability to modulate learning and cognition significantly, it appears to be a promising technique for speech rehabilitation such as in cases of post-stroke aphasia.^3^ In fact, animal and mechanistic human studies have confirmed the notion that transcranial direct current stimulation induces significant and lasting local neuroplastic changes.^4^ ^,^ ^5^

In transcranial direct current stimulation, cortical tissues are polarized by a constant electric current field applied via two electrodes placed on certain areas of the scalp.^6^ ^,^ ^7^ Two previous transcranial direct current stimulation studies on cases of chronic aphasia have shown beneficial results. In these studies, transcranial direct current stimulation was applied to the left prefrontal cortex, targeting Broca's area. These studies showed that patients who received active transcranial direct current stimulation presented improvements in naming abilities, in comparison with sham transcranial direct current stimulation.^8^ ^,^ ^9^ Other studies among healthy subjects have also shown that transcranial direct current stimulation can have positive effects on language skills.^10^ ^,^ ^11^

The contribution of the primary motor cortex to language has been shown by neuroimaging studies,^12^ and previous studies showing that modulation of the unaffected motor cortex is an advantageous strategy for decreasing imbalanced interhemispheric activity in stroke cases.^13^ ^,^ ^14^ Based on these data, our aim was to conduct a preliminary open-label study to assess the effects from modulating plasticity by means of excitability-diminishing cathodal transcranial direct current stimulation over the unaffected primary motor cortex, in order to obtain preliminary efficacy and safety data on its effects on language.

## OBJECTIVE

The purpose of collecting this data was to describe a cohort of patients who were aphasic after stroke and recovered through transcranial direct current stimulation. The objective of the study was to explore the effects and feasibility of transcranial direct anodic current stimulation of the uninjured primary motor cortex among patients with aphasia after stroke, with regard to language rehabilitation.

## METHODS

We studied 19 ischemic stroke patients (9 men and 10 women) with a mean age of 53.3 years, at least 6 months after their strokes. Table 1 provides additional demographic information. All patients had suffered left-hemisphere injury caused by the stroke. Stroke was defined as an ischemic focal neurological deficit that persisted for at least 24 hours. The diagnosis was made using aphasia classification of clinical features and confirmed by means of neuroimaging studies. The lesions were located in the frontal lobe, parietal lobe, temporal lobe and subcortical areas. We excluded patients with any clinically significant or unstable medical or psychiatric disorder, history of substance abuse, any neuropsychiatric comorbidity other than stroke and contraindications for transcranial direct current stimulation. Written informed consent was obtained from all participants prior to inclusion in the study, which was approved by the local ethics committee, under number 375/07. The aphasia classification was based on Hedge,^15^ Mac-Kay et al.^16^ and Ortiz,^17^ and comorbidities such as dysarthria or apraxia of speech were excluded.

**Table 1:** Patients' characteristics

Patient	Gender	Age (years)	Aphasia type
P1	female	74	Mixed
P2	female	61	Broca
P3	female	65	Anomic
P4	female	51	Broca
P5	female	70	Mixed
P6	female	50	Mixed
P7	female	22	Broca
P8	female	60	Broca
P9	female	48	Broca
P10	female	25	Mixed
P11	male	69	Broca
P12	male	54	Anomic
P13	male	63	Anomic
P14	male	33	Anomic
P15	male	53	Broca
P16	male	54	Anomic
P17	male	62	Anomic
P18	male	56	Broca
P19	male	42	Anomic

Direct current was transferred through a saline-soaked pair of surface sponge electrodes (35 cm^2^) and was delivered by means of a specially developed direct current stimulator with a maximum output of 10 mA. To stimulate the primary motor cortex (M1), one electrode (cathode) was placed over C3/C4 (International 10/20 Electroencephalogram System), which corresponded approximately to the location of the primary motor cortex of the unaffected side, and the other electrode (anode) was placed on the contralateral supraorbital area. Transcranial direct current stimulation was applied for 20 minutes at a current intensity of 2 mA, on 10 consecutive days (with an interval during weekend days).

Language assessment was performed before and after each session using three language test batteries: the Montreal Toulouse battery (alpha version),^18^ the Boston naming test^19^ and the Verbal Fluency Test.^20^

The Montreal Toulouse battery consists of the following tasks: guided interview; oral comprehension using words, simple phrases and complex phrases; written comprehension using words, simple phrases and complex phrases; copying of written phrases; word and phrase dictation; reading; repetition; and naming. These specific tests were selected because they had previously been validated for Brazilian Portuguese and had been shown to provide reliable data in speech pathology investigations, as shown by Santos et al.^21^ and Mac-Kay.^16^

The Verbal Fluency Test consists of asking the patients to name animals, using words with the phonemes /f/ and /s/ within one minute. This test is part of the evaluation of the Consortium to Establish a Registry for Alzheimer Disease (CERAD),^22^ which was adapted for use in Brazil by Bertolucci et al.^20^

The patients received the neuromodulation in a silent and well-lit room, and the responses to the tests were noted down on the Montreal Toulouse, Verbal Fluency and Boston Naming answer sheets. Furthermore, all the participants were instructed not to undergo any speech therapy during the period of the tests so that there would not be any interference in the results.

Since this was a pilot study with only one stimulation group, we conducted an exploratory analysis in which we compared the scores before and after stimulation. Moreover, we did not correct by means of multiple comparisons because this was a small pilot study and the main aim was to explore the best outcomes for further confirmatory trials. Since the data were not normally distributed in some of the outcomes, we used the Wilcoxon signed rank test for all testing. The data were reported as means and standard deviations. Statistical significance was taken to be a two-tailed P-value of < 0.05.

## RESULTS

All the patients received active transcranial direct current stimulation, with no adverse effects registered. The patients tolerated the treatment well. Only one patient who had been selected abandoned the study because of hospitalization due to pneumonia and clinical respiratory complications. This patient underwent only one stimulation session. No significant adverse effects from applying brain stimulation to these stroke patients were reported. Table 2 shows the mean performance before and after stimulation for the assessments that we measured in our study, together with the statistical results.

**Table 2:** Performance in the language assessments before and after transcranial direct current stimulation (tDCS)

TASK	Before tDCS mean	After tDCS mean	Before tDCS sd	After tDCS sd	P-value
Interview	4.00	4.27	4.27	4.17	0.180
WC	3.36	3.91	2.014	1.64	0.084
SPC	1.82	2.36	1.33	1.03	0.034
CPC	1.55	1.46	1.21	1.21	0.739
WWC	3.18	3.18	1.78	1.89	1.00
WSPC	1.55	1.64	1.37	1.43	0.564
WCPC	1.09	1.36	0.94	1.21	0.180
Copying	0.55	0.64	0.52	0.51	0.317
Dictation	0.82	1.00	1.40	1.61	0.157
Word reading	2.55	2.91	3.56	3.36	0.157
Phrase reading	0.64	0.64	1.21	1.12	1.00
Word repetition	3.91	4.00	3.36	3.29	0.783
Phrase repetition	0.36	0.46	0.92	0.69	0.564
Naming	4.18	5.18	5.33	5.00	0.041
Verbal fluency animals	3.45	4.18	5.20	5.91	0.038
Verbal fluency S	1.18	2.09	2.27	3.17	0.068
Verbal fluency F	2.00	2.55	3.44	4.08	0.131
Boston naming test	3.45	4.27	4.82	5.62	0.056

WC = word comprehension

SPC = simple phrase comprehension

CPC = complex phrase comprehension

WWC = written word comprehension

WSPC = written simple phrase comprehension

WCPC = written complex phrase comprehension

sd = standard deviation

S = words starting with the phoneme /s/

F = words starting with the phoneme /f/

Statistically significant differences from before to after the sessions were only found in relation to simple phrase comprehension (P = 0.034), naming (P = 0.041) and verbal fluency in the animal tasks (P = 0.038). An increase in the number of correct answers after stimulation was observed for the Montreal Toulouse battery (alpha version). The other outcomes did not yield any significant results (see Table 2 for additional details).

Table 1 shows the characteristics of the patients: schooling, gender, age, injured hemisphere, year of stroke and aphasia type. 

## DISCUSSION

The results from this pilot study showed that transcranial direct current stimulation was well tolerated and that cathodal transcranial direct current stimulation of the unaffected primary motor cortex was associated with significant improvements in the following language tasks: simple phrase comprehension, naming and verbal fluency in relation to names of animals.

One topic that needs to be discussed is our rationale for motor cortex stimulation. We chose to modulate motor cortex plasticity because there is an important link between motor cortex activation and language processing that suggests that these two systems (language and motor) have common neural networks. For instance, a recent study showed that preactivation of the leg motor cortex with patients standing, in comparison with sitting, was associated with increased performance in a picture-naming task among patients with aphasia.^23^ Other studies have also confirmed that the motor cortex has a role in language recovery following aphasia.^12^ ^,^ ^24^

In this context, we hypothesized that motor cortex modulation would be an interesting target for language recovery treatment. We chose the unaffected motor cortex, based on previous experience showing that this is an effective target for enhancing motor recovery in stroke cases. In fact, previous studies have shown that transcranial direct current stimulation applied in the area homologous to the lesion in the unaffected hemisphere can be a good strategy for reverting increased transcallosal inhibition of the affected motor cortex in stroke cases.^25^ Other transcranial direct current stimulation studies have described improvements in function post-stroke, with induced modification of excessive interhemispheric inhibition.^26^ ^,^ ^27^

One significant limitation to the present study is that we used a large electrode (35 cm^2^) over the motor cortex, thereby causing adjacent areas, such as the premotor areas and posterior areas, including the somatosensory cortex, to be stimulated in addition to the main target area. Additionally, the effects can also be attributed to the contralateral anodal electrode over the supraorbital area. Because it is expected that activity in this area would be increased with this electrode, it is possible that modulation of the right orbitofrontal cortex was involved in the positive effects on language improvement. Further studies using larger reference electrodes or even using high-density transcranial direct current stimulation over the unaffected hemisphere need to be conducted in order to detangle the effects observed in the present study.^28^ Finally, because of the lack of control group, it is possible that the improvement observed in this study was due to a placebo effect. However, the improvement in some of the tests had a large effect size, and this is less likely to be explained by a placebo effect alone. Another point to note is the heterogeneity of aphasia types observed. The mixed aphasic patients included in this study had minimal comprehension deficit with regard to answers in the language tests. Hence, these patients showed more evidence of expressive impairment than of comprehensive difficulties. 

It is essential to compare our results with other transcranial direct current stimulation studies. Baker et al.^9^ tested transcranial direct current stimulation in ten patients, with the aim of treating aphasia after stroke. Anodal transcranial direct current stimulation (active or sham) was placed over the left frontal cortex and the cathode was placed on the contralateral area for five consecutive days, using the parameter of 1 mA for 20 minutes. The authors found that anodal transcranial direct current stimulation over the left frontal cortex improved naming abilities among stroke patients with aphasia. Monti et al.^8^ also observed language improvement, but with a different study design: they tested transcranial direct current stimulation on eight patients (anodal transcranial direct current stimulation, cathodal stimulation and sham over Broca's region, with one-week intervals between sessions), using 2 mA for 10 minutes of stimulation in each session. In their study, it was shown that anodal transcranial direct current stimulation and sham transcranial direct current stimulation did not induce any significant changes, whereas cathodal transcranial direct current stimulation significantly improved performance in the picture naming task, by a mean of 33.6%. Therefore, these two previous studies showed positive results from transcranial direct current stimulation over left frontal areas. Based on these results, one alternative explanation for our results is that our reference electrode over the prefrontal cortex was responsible for some of the effects seen here. However, in our study, the anodal electrode was placed over the right supraorbital area (instead of the left area as in the other studies).

## CONCLUSIONS

The present study demonstrated significant language task improvement after cathodal transcranial direct current stimulation of the unaffected motor cortex. It gives additional support to the initial studies showing beneficial effects from transcranial direct current stimulation in relation to language recovery and provides support for further studies. Additional sham-controlled trials and also trials assessing alternative electrode montages are necessary in order to investigate the role of motor cortex modulation for treating of aphasia by means of transcranial direct current stimulation.
